# Instruments and Technic to Be Used in Pulp Canal Operations*Read before Michigan State Dental Society and reprinted in abstract from the February, 1919, *Journal N. D. A*.

**Published:** 1919-03

**Authors:** Elmer S. Best


### INSTRUMENTS AND TECHNIC TO BE USED IN PULP CANAL OPERATIONS*

BY ELMER S. BEST, D.D.S.

In presenting this subject, the writer is not attempting to show upon what teeth and under what conditions pulp canal operations are indicated. The incorporation of this phase of the subject would make the paper entirely too cumbersome; hence, it is omitted. However, it is of some importance that a little light be cast on this very important part of our work, and the writer wishes to state that in his opinion certain conditions should prevail, viz:

1. Infected areas surrounding non-vital teeth are a distinct menace and should be removed.
2. A large number of non-vital teeth are being extracted.
3. Very few vital pulps will be removed in the future.
4. Due thought should be given to the strategic value of certain non-vital teeth when removing a large number of such teeth.
5. Never attempt to do a pulp canal operation unless prepared and equipped to do it thoroughly and aseptically.
6. Under favorable conditions pulp canal operations are distinctly indicated, and we can proceed with our work with a reasonable assurance of success.

### STEPS OF PULP CANAL OPERATIONS FOR VITAL TEETH

Apply local anesthetic and remove decay, but do not enter pulp chamber.

Spray mouth and paint gums with iodin, where dam is to be applied.

Apply dam and tincture of Green soap, water, iodin and alcohol to crowns of teeth included in dam.

* Read before Michigan State Dental Society and reprinted in abstract from the February, 1919, *Journal N. D. A.*

Apply 1% Chlorazene to cavity on pledget of cotton and open pulp chamber, cut away liberally.

Enter large canal with barbed broach and try to remove pulp.

Absorb hemorrhage resulting and place dry absorbent point in canal and temporarily close with temporary stopping.

In small canals:

Try xxx Barbed Broach and if canal will accommodate it try to remove pulp en masse.

If xxx Barb is too large, try xxx short barb and then the apex curette.

If none of these will pass into canal, use the pathfinder. Work it carefully into the canal, apply 30% H2 SO4 and by a stirring motion, press back the walls of the canal as they are softened by the acid, and follow with sodium bicarbonatic.

Now use short barbed and file walls of the canal with broaches, using larger sizes of short barbed broach.

Sometimes Kerr file is indicated to open a constriction in a canal.

Use apex curette and work faithfully and diligently, removing debris from apical portion of the canal.

With Kerr files and short barbed broaches enlarge canal to required size. Seal a mild dressing with oxychloride of zinc cement and dismiss patient. Caution patient to not bite on tooth for fear of splitting the crown.

At next appointment use measurement wires, ray the tooth and fill canals if ready.

STEPS OF PULP CANAL OPERATIONS FOR NON-VITAL TEETH

Make radiographic examination of tooth to be operated upon, and test vitality with battery.

Remove decay and open pulp chamber. Wash out freely, spray mouth, and paint gums where dam is to be applied with iodin.

Apply dam and wash with tincture of Green soap, water, iodin and alcohol successively.

Apply 1% chlorazene to cavity on cotton.

With barbed broaches or apex curettes remove contents of canal for about one-third the length of the canal.

If canals contain guttapercha cones, remove with Xylol.

Seal in each canal independently with oxychloride of zinc cement and formocresol.

Moisten walls of cavity with Xylol, fill cavity with copper stopping. Caution patient to not bite on tooth for fear of fracturing it.

Next sitting, remove dressing and wash out with normal salt solution. Use Sodium Potassium and cleanse canal, but do not enter apical third. Place dressing of 1% chlorazene for a few moments. Dry out and if the peridental membrane is broken as shown in radiograph, seal formocresol in apical portion. Otherwise use oil of cloves, unless a pronounced odor of putrifaction is still present, in which event do not hesitate to use formocresol.

Use measurement wires and ray.

Next sitting clean out apical portion with apex curettes. Ionize, and if free from bacteria seal the foramina and fill the canal.

NOTE: In view of the responsibility that rests upon the operator in retaining teeth that have been pulpless for some time, it becomes a matter of necessity that careful bacteriological examinations be made of all such canals and they must not be filled until they are bacteria free.

### FILLING THE CANALS

In the event of the peridental membrane being destroyed, a limited amount of encapsulation is indicated but it is not indicated when a vital pulp has been removed.

In filling canals four methods can be employed.

1. Chlorapercha and guttapercha cones.
2. Rosin and chloroform, placing the solution in the canal before we do the cone and pumping the cone up and down in the canal until dissolved.

3. Placing a cone that goes about to the apex in the canal first. Then placing Rosin Chloroform solution in along side the cone. Withdraw cone slightly, cut off and remove as much of the cone as possible and with fine canal pluggers condense the cone in the apical portion. This is many times the best method to employ.

4. Cutting off a piece of a cone and placing it on the end of a carefully measured canal plugger and deftly placing it at the end of the root.

### RUBBER DAM CLAMPS AND BURS

These are kept in a small petrie dish containing equal parts of alcohol and glycerine.

### ABSORBENT POINTS

There are two methods of keeping these points. They are wrapped in 6 x 6 gauze and sterilized, or are placed in glass jars and sterilized.

### PULP CANAL APPLICATORS

These instruments mounted in aluminum handles are for the purpose of absorbing moisture from the canal or wiping out a canal. The cotton on these instruments is not left in the canal as part of a dressing. These instruments must be wrapped in such a manner that the cotton is locked on the instrument and will not loosen during the process of sterilization. The material of which the instrument is made must be of such a nature that it will not rust and yet must be very fine. The tantalum instrument of the Twentieth Century Dental Broach Company and the three nonrust canal probes manufactured by the Detroit Dental Manufacturing Company are paruicularly suited to this work. In wrapping the instrument, a small piece of cotton is placed between the thumb and first finger of the left hand. Then the instrument is laid on the cotton and seized with finger and thumb. Some of the cotton is left projecting, and this is now seized with the finger and thumb of the right hand and together with the instrument is rolled. This locks the cotton at both ends. The instruments in one-half dozen lots are wrapped up in 6 x 6 gauze, and put through the sterilizer. Upon the completion of sterilization they are removed and placed in a glass jar. ready for use.

### SEALING DRESSINGS

Gain access to all canals and seal dressing in each canal independently with oxychloride of zinc cement.

Moisten walls of cavity with Xylol and fill with temporary stopping.

### CHLOROFORM AND ROSIN SOLUTION

Four grains rosin.

Three drachms chloroform.

In making this preparation use the rosin that is used by violin players on their bows. Care must be taken that the solution does not become too thick due to the evaporation of the chloroform.

### CHLORAPERCHA

White base plate guttapercha is cut in one-half inch squares and put in alcohol for about one hour. Then placed in a receptacle and covered with chloroform. When dissolved, strain through gauze into permanent receptacle. Care must be taken to not allow the solution to become too thick.

### MEASUREMENT WIRES

The use of measurement or diagnostic wires is quite essential in efficient root canal work. In the first place, it is quite impossible for us to know how near we are to the apex while operating, and one of the most serious mistakes we can make is to pass prematurely through a foramen carrying with our instrument, infectious material Great stress should be laid on this point.

In the event of the patient feeling pain in a tooth while operating we are at a loss to know whether it is pulp tissue or peridental membrane. Place a measurement wire in the canal and ray the case. The end of the wire being at the point of sensation we can easily tell when we see our film whether or not we were still operating in the canal or were through the root end.

### XYLOL

It is a synthetic preparation and is used for the purpose of dissolving old guttapercha canal fillings. It is applied to the guttapercha, allowed to remain for a few moments, and then the guttapercha is worked out with Rhein pics or Kerr reamers.

### ROOT CANAL SYRINGES

The canal should be frequently washed out but care must be taken not to force any solution through the canal into the periapical area, and that no moisture remains in the canal while operating. In the latter event, the moisture, during our instrumentation, will be slowly worked through the apex. It is a useful and efficient instrument in skilled hands, but is capable of doing a great amount of damage.

### TRANSILLUMINATING LAMP

Sometimes when we have lost the course of a canal, apply a small drop of iodin, allow it to remain for a few moments and then remove it with alcohol. A small amount will penetrate the unopened canal and by placing the transilluminating lamp along side the tooth it will so illuminate the inside that we can easily locate the tiny dark spot which is the entrance to the canal. Then by placing a very rigid instrument on this spot we can force our way into the remainder of the canal.

### ROOT CANAL INSTRUMENTS

Instruments should be made ready for the root canal operation. The instruments actually used in the canal and those which are used outside the canals and which soon become contaminated should be kept separated.

### COTTON PELLETS

The Richmond pellets for this purpose are prepared for use by placing in small petrie dishes or in 6 x 6 gauze.

### GUTTAPERCHA POINTS

Are flattened to facilitate grasping with pliers. This can be done with a pair of pliers with serrated beaks.

### PULP CHAMBER BURS

In opening a pulp chamber we want a bur that has a long cutting surface. It should not cut on the end as we must be careful not to mutilate the floor of the pulp chamber. Mounted stones serve nicely in smoothing up the walls.

While operating in root canals, we should simplify our work as much as possible by funneling the openings to the canals, as this will facilitate very greatly the passing of instruments, dressings and guttapercha points into the canals. It can be performed with flame shaped burs or with a hand instrument.

### WILMONT-CASTLE STERILIZER

In using this sterilizer, operate it in such a manner that the warm air passes through the sterilizing chamber for half an hour, then superheated steam for half an hour, then dry warm air again for half an hour.

A simple little sterilizer for pulp canal instruments. This is made with a four-inch electric plate, a two-inch band of brass soldered and set over the plate and a four-inch porcelain dish set in the brass band. Have this band plated and the sterilizer presents a very creditable appearance.

Cotton pellets and Mayo sponges used in curettements and resections. The Mayo sponges, which are made in many sizes, will be found to be very valuable.

### THE BARBED BROACH

Up to within the past four or five years, the instrument which we chiefly depended upon for removing the dental pulp was the ordinary barbed broach. This instrument so little understood, has been the cause of more trouble in this work than any of us have any conception. If a canal was large enough to accommodate the size generally used, the pulp was removed. If it was not, we went as far as we could and stopped. The instrument was, in the main, too large to be of any use in any but the large canals. Most frequently arsenic had been used, and the pulp tissue in the finer canals was relieved of its vitality, so the contents of such canals were as a general rule left undisturbed.

In the manufacture of this instrument in the finer sizes such as the xxx and xx fine, which instruments have made their appearance in considerable quantities, we find that a large percentage are defective. Hence, in purchasing these instruments, beware of a brand that is cheap, as it is in all liklihood the discards or defectives you are getting.

The xxx and xx fine barbed broaches have made possible the removal of organic tissue from very much smaller canals than had heretofore been possible. The instrument is made in a variety of styles:

1. The more common unmounted type.
2. Mounted in long aluminum handles.
3. The Midget Barbed mounted in aluminum handles.
4. The Short Thumb Broaches for use in posterior teeth.
5. The Right Angle Broach for use in the right angle broach holder.

### THE SHORT BARBED BROACH

The need of such an instrument has been felt for a long time and its advent means a great deal to operators while working in pulp canals.

It has been realized for some time that one of the most efficient instruments used in enlarging canals is the barbed broach, but on account of the comparatively large diameter of a barbed broach its use in fine canals has not been very extensive.

The Short Barbed Broach possesses many of the advantages of the ordinary barbed broach, and in addition has such a reduced diameter that its use in fine canals is made possible. It is used solely for the purpose of filing out the walls of the canal and is not used for removing organic tissue from the canal. In using the instrument it is carefully inserted into the canal and withdrawn against the canal wall, exerting as much pressure as possible laterally. Never attempt to rotate or twist this instrument in a canal. It is not intended for such a purpose and if put to such use, is liable to break and leave a portion in the canal.

The most suitable long handle type is the Midget, as in this instrument the length of the wire is reduced considerably and gives us greater control over the cutting part of the broach.

The Short Barbed Broach comes in various sizes, from xxx fine up to coarse. By simply increasing the size of the instruments we are able to enlarge the canal almost at will.

The instrument is also made for right angle use and is so constructed that it fits the Ivory Right Angle Broach Holder.

### APEX BROACH

Is a combination barbed and smooth broach. At the extreme tip are placed two or three barbs. It is the presence of these barbs that gives the instrument its operating value. The apex broach is made in three styles: the regular long handle type, the midget, and the right angle.

This instrument has done more than any other instrument to reveal to the operator how far he has come from completing his task of cleansing the canals. After all the other instruments have been used, this instrument slipped into the canal and operated in the apical portion of the canal will bring forth shreds of cotton, small pulp nodules, fibers of pulp tissue and several unrecognizable types of debris which, had any been left in the canal, would have been forced through the foramina during the process of filling the canal and deposited in the periapical tissues to set up a condition resulting in a periapical infection.

### THE FINE SMOOTH BROACH

Until about at the same time that the barbed broaches were changed, due to strong pressure brought to bear on the manufacturer, the smooth broach was generally used for wrapping cotton, for drying the canal or placing a dressing in the canal. However, a new use was found for it. It is used for tracing out or locating fine canals. For this purpose it is without an equal. Its very fineness makes it possible for us to insert this instrument into a canal and by cautiously pushing, turning and twisting it, we are very agreeably surprised in countless instances to find that we have reached the apex. When we have done so, we place a drop of 30% H2 SO4 or Phenol Sulphonic Acid at the orifice of the canal, and by a stirring motion we carry the drug around the instrument into the canal. The drug attacks the walls of the canal and by our circular motion we push back the dissolved portion and thereby enlarge the canal.

Many times we are unable to locate a lost canal and trace it to the foramin. A very simple but extremely efficient method, is to slightly bend the broach about 2

or 3 millimeters from the point and with this little hook we search out the lost canal.

Many times we can see in our preliminary radiograph that the root curves in a certain direction, and by placing the point of the instrument in that direction we frequently slip into the canal and continue our operations. In the mesial roots of lower molars in case we are working in the buccal canal and apparently meet with an obstruction, by simply turning the point to the lingual, we will probably locate the canal, similarly in case we are working in the lingual canal it may turn to the buccal, and it is in this direction we find the canal.

### SODIUM POTASSIUM

The greatest care should be exercised in handling this material. Do not allow the small tube in which the material is kept to come in contact with water. Do not force any blunt instrument into the tube. Seal the tube with warmed wax carefully moulded over the end when finished. To reach the material when considerable has been used, take a file and file around the tube and break it off. The Sodium Potassium is carried to the canal preferably on Young broaches.

